# Characterization of the Ayran Made with Commercial Probiotic Cultures for Fatty Acids, Cholesterol, Folic Acid Levels, and Anti-Oxidative Potential

**DOI:** 10.1007/s12602-023-10100-7

**Published:** 2023-06-06

**Authors:** Ola M. A. K. Shalabi, Amina M. Hassan, Magdy M. Ismail, Reham K. El-Menawy

**Affiliations:** 1 Dairy Department, Faculty of Agriculture, Mansoura University, Mansoura, Egypt; 2 Dairy Technology Research Department, Animal Production Research Institute, Agricultural Research Center, Dokki, Giza, Egypt

**Keywords:** Ayran, Probiotic, Cholesterol, Antioxidant activity, Folic acid

## Abstract

Ayran is a salted drinkable fermented milk food which consumed in many countries around the world. In this study, some chemical parameters were determined to evaluate the healthy properties of ayran prepared using various commercial probiotic cultures. Four treatments of ayran were made from cow’s milk and using classic yogurt culture (*L. delbrueckii subsp. bulgaricus* and *Streptococcus thermophilus*) [T1], ABT-5 culture (*L. acidophilus, Bifidobacterium* and *S. thermophilus*) [T2], exopolysaccharide producing culture (EPS-producing, *L. delbrueckii subsp. bulgaricus* and *S. thermophilus*) [T3], and EPS-producing culture + *Bifidobacterium animalis* subsp. *lactis* BB12 (mixture culture) [T4]. Treatment 1 had the highest acidity, acetaldehyde, and diacetyl values. Using probiotic [T2] or mixture cultures [T4] reduced saturated fatty acids by 1.97% and increased monounsaturated and polyunsaturated fatty acids of ayran by 4.94 and 5.72%, respectively. Also, the levels of oleic acid (omega-9), linoleic acid (omega-6), and α-linolenic acid (omega-3) increased in ayran produced using probiotic or mixture cultures. Sample T4 was highly richer in the value of antioxidant activity (27.62%) and folic acid (0.1566 mg/100 g) whereas possessed the lowest cholesterol amount (8.983 mg/100 g). Mixture culture (EPS-producing culture + *Bifidobacterium animalis* subsp. *lactis* BB12) is a good starter to improve the healthy and nutritional characteristics of bio-ayran.

## Introduction

A fermented milk beverage called ayran is prepared from either whole or skim milk (cow, goat, and sheep). Various names, such as “lassi” in India, “doogh” in Iran, “Tan” in Armenia, “Shenina” in Jordan, “Laban Ayran” in Syria and Lebanon, “Laban Arbil” in Iraq, “Ayrani” in Cyprus, and “ayran” in Turkey, are used to name to it in various countries. Traditional and industrial methods are used to prepare ayran. The traditional method for preparing ayran involves continuously stirring yogurt with added water and salt for 1–2 min at room temperature. The industrial process requires adding water to milk to standardize the total solids content to about 8%. Ayran is produced by fermenting standardized milk at 42 °C with yogurt culture (*Lactobacillus delbrueckii subsp. bulgaricus* and *Streptococcus thermophilus*) until the value of pH reaches 4.2–4.6. The salt (0.5%) is then added and stirred. Ayran is a highly valued, easily digestible beverage with a high vitamin and calcium content. It indicates that ayran, which presently has advantageous properties, will be able to develop functional attributes [1].

On the other hand, for many years, fermented dairy products have been widely produced using lactic acid bacteria. Several chemical, rheological, and functional changes occur when lactic acid bacteria ferment milk. For instance, using *Streptococcus thermophilus* and *Lactobacillus delbrueckii subsp. bulgaricus* to prepare yogurt from fresh goat milk, some changes were noticed in the profiles of certain fatty acids [2]. According to other clinical investigations, *Bifidobacterium animalis sp. lactis* BB-12, alone or in combination with other probiotics or ingredients, increases the number of beneficial bacteria while decreases the population of possibly pathogenic bacteria [3]. Ejtahed et al. [4] showed that probiotic yogurt containing BB12 has beneficial effects on metabolism, including lowering LDL cholesterol in type 2 diabetic patients, increasing HDL cholesterol in adult women, and improving glucose tolerance during pregnancy [5].

Several lactic acid bacteria (LAB) produce extracellular polysaccharides, called exopolysaccharides (EPS), that enhance rheological, physical, and sensory properties of foods made from fermented milk. Behare et al. [6] showed that utilization of EPS-producing *Streptococcus thermophilus* IG16 led to lassi with the ideal amount of acidity, little syneresis, high viscosity, and higher ratings for consistency, flavor, color, and appearance. Yilmaz et al. [7] cleared that a novel technique that can significantly enhance the organoleptic and technological properties of fermented dairy products like ayran is the selection of EPS-producing cultures to increase texture and viscosity. In addition to improving the food’s technological qualities, some EPS produced by LAB showed positive benefits on human health [8]. There are scientific studies that EPS, which are biopolymers with a wide variety of biological activities and technological characteristics, are beneficial to the health [9]. Different EPS from LAB have been evaluated for their antibacterial activity in vitro and in vivo against a variety of pathogenic microorganisms. EPS can act as an indirect antimicrobial agent by (i) activating the innate and adaptive immune response or (ii) promoting the growth and/or formation of biofilms of other advantageous commensal bacteria or probiotics [10]. There have been numerous studies on the effects of milk, culture type, and processing conditions on the rheological and chemical properties of ayran, but none have studied at the impact of culture type on ayran’s health-related characteristics. Also, no extensive studies about the impact of utilization of mixed culture (EPS producing + probiotic culture) on the healthy and nutritional properties of ayran are found. Subsequently, the main aim of this study is to evaluate the concentrations of free fatty acids, cholesterol, aroma compounds, antioxidant activity, and folic acid as indicators of the quality and healthy properties of ayran made using different starters.

## Materials and Methods

### Materials

Fresh raw cow milk (acidity 0.18%, pH 6.60, total solids 13.23, fat 4.3, and total protein 3.68%) purchased from the farm of Animal Production Research Institute, Giza, Egypt, was used for preparing ayran. Direct vat set (DVS) commercial cultures of classic yogurt (*L. delbrueckii subsp. bulgaricus* and *Streptococcus thermophilus*, 1:1), ABT-5 (*L. acidophilus, Bifidobacterium* and *S. thermophilus*), YO-Flex Mild 1.0 (EPS-producing, *L. delbrueckii subsp. bulgaricus* and *S. thermophilus*), and BB-12 (*Bifidobacterium animalis* subsp. *lactis*) were procured from Chr. Hansen’s Lab A/S Copenhagen, Denmark.

### Methods

#### Ayran Preparation

First, 80 L of cow milk were pasteurized at 90 °C for 15 min, cooled to 40–42 °C, and then divided into four treatments (20 L/treatment). The various cultures were added at a 1% concentration as follows:

- Treatment [T1]: classic yogurt culture (*L. delbrueckii subsp. bulgaricus* and *Streptococcus thermophilus* YoFlex^®^ Premium 4.0, 1:1). The bacterial strains contained 7.7 and 7.4 Log10 CFU/gm of freeze-dried culture, respectively.
- Treatment [T2]: ABT-5 culture (*L. acidophilus* La-5, *Bifidobacterium* spp., and *S. thermophilus* TH-4, 1:1:1). The bacterial strains contained 8.2, 9.5, and 7.3 Log10 CFU/gm of freeze-dried culture, respectively.
- Treatment [T3]: YO-Flex Mild 1.0 (EPS-producing) culture (*L. delbrueckii subsp. bulgaricus* and *S. thermophilus*, 1:1). The bacterial strains contained 7.8 and 7.3Log10 CFU/gm of freeze-dried culture, respectively.
- Treatment [T4]: YO-Flex Mild 1.0 (EPS-producing) culture + *Bifidobacterium animalis* subsp. *lactis*, BB-12 (1:1:1). The bacterial strains contained 7.8, 7.3, and 9.4 Log10 CFU/gm of freeze-dried culture, respectively.

After inoculation, the samples of T1, T3, and T4 were incubated at 42 °C for 3 h, whereas the sample of T2 was incubated at 40 °C for 4 h. The ayran treatments were made by adding salt solution (1%) to the curd of each treatment (60% curd: 40% salt solution). The salt solution was heated at 85 °C for 10 min for pasteurization then cooled to room temperature and mixed with the curds. The four mixtures (curd and salt solution) were homogenized by using a mixer for 3 min at 20 °C and 3000 rpm. Various ayran samples were stored at 4 °C for 14 days and analyzed when fresh and after 7 and 14 days of refrigerated storage.

#### Chemical Analyses

According to the Association of Official Analytical Chemists (AOAC) [11] methods, the titratable acidity and moisture of ayran samples were determined. Acetaldehyde and diacetyl contents were determined according to Lees and Jago [12]. Using the stable radical DPPH, the antioxidant activity of ayran milk was evaluated in terms of its hydrogen-donating or radical-scavenging activity, as reported by Politeo et al. [13]. DPPH was used as a stable radical. A volume of 2 mL of DPPH in ethanol (500 mM) was added to 2 mL of the whey fraction of different ayran samples, mixed vigorously, and allowed to stand in the dark at room temperature for 30 min. The absorbance was measured at 517 nm. Ethanol was used as a blank, while DPPH solution in ethanol served as the control. The radical scavenging activity of the samples was expressed as % inhibition of DPPH absorbance:

$$\text{Inhibition}=[A\text{control}-A\text{test}/A{\text{control}}]\times 100$$

where *A*control is the absorbance of the control sample (DPPH solution without whey fraction) and *A*test is the absorbance of the test sample (DPPH solution plus whey fraction).

#### Fatty Acid Analysis

##### Sample Preparation

Identification and quantification of free fatty acids were performed according to the method described by Jahreis et al. [14]. Fat from 4 g ayran was extracted using 15 ml of Fokh’s reagent (chloroform/ methanol = 2:1 (v/v)). The extracted lipids were filtered over anhydrous Na_2_S0_4_. Fatty acids methyl ester (FAMEs) was prepared by transesterification with potassium methylate. 0.5 ml potassium methylate (5% wt/wt in methanol) was added to the fat solution in the Pyrex@ tube. The tube was tightly capped, vortexed, and heated at 60 °C for 15 min in a drying cabinet. After cooling down, 1.5 ml sulfuric acid (2% wt/wt in aq. dest.) was added, and the tube was vortexed again. 1 µl from the clear organic phase was injected into the gas chromatograph.

##### Gas Chromatography (GC) Analysis

According to the method described by Jahreis et al. [14], free fatty acid identification and quantification were performed. Fatty acids were determined by gas–liquid chromatography using a ZB-5 fused silica capillary column, and 1 µl of FAMEs was injected into a GC-MS autosampler (7890 A GC System Agilent) fitted with an MSD detector. To identify the fatty acids, the internal standard, methyl tricosanoate (23:0) from Sigma, was used to quantify the fatty acids in mg g^−1^ total lipids. Before transesterification, all samples received 1.00 mL of internal standard solution (1 mg mL ^−1^), and the solvent was evaporated under N2 flow.

#### Folic Acid Analysis

From Sigma-Aldrich (St. Louis, MO, USA), a reference standard for folic acid (CAS No. 59-30-3) was purchased. The fresh ayran samples were prepared as described by Gujska et al. [15]. According to Wu et al. [16], folic acid was separated chromatographically using HPLC on a Zorbax Eclipse XDB-C18 [4.6 × 250 mm, Agilent Technologies, Santa Clara, CA] at 280 nm. Mobile phase A consisted of 20% H_3_PO_4_ in water (vol/vol, pH 7.2), phase B consisted of 100% methanol (vol/vol), and the ratio of A to B was 90:10 under a flow rate of 1 mL/min.

#### Cholesterol Analysis

The analysis of cholesterol content in fresh ayran samples occurred according to Kolaric and Šimko [17]. A total of 5.0 g of samples were refluxed with 0.015 L of 1 mol.L^−1^ KOH methanolic solution for 15 min. The extraction process was performed with the 0.015 L of the extraction solvent composed of n-hexane and chloroform (1:1, v/v) in duplicate. The extracts were filtrated through anhydrous Na2SO4 and evaporated using a rotary vacuum evaporator. The residue was then dissolved in 5 mL of methanol, filtered using a syringe PTFE filter with 0.2 μm membrane (Agilent Technologies, USA), and analyzed by HPLC. All procedures were triplicated.

#### Statistical Analysis

The SPSS 17.0 program was used to do an analysis of variance (ANOVA). Using Duncan’s multiple range test (*p* < 0.05), significant differences among samples were determined.

## Results and Discussion

### Acidity and Moisture Contents of Ayran

Data of the impact of using various cultures in ayran production on the acidity and moisture values during the storage period are depicted in Table 1. Ayran’s titratable acidity values differed significantly (*P* < 0.05) among the samples. Ayran made using classic starter [T1] exhibited the highest acid production, whereas probiotic ayran [T2] possessed the lowest one. These findings were supported by the findings of Shihata and Shah [18] but contrasted with those of Kehagias et al. [19]. According to Shihata and Shah [18], ABT starters are known to produce yogurt with a good, mild taste and low post-acidification, whereas Kehagias et al. [19] mentioned that *B. bifidum* produces acetic and lactic acids, which causes increasing the yogurt acidity. However, Ayar and Burucu [20] revealed that the acidity values of ayran manufacture using classic yogurt culture, *L. acidophilus*, or classic yogurt culture + *L. acidophilus* were 0.227, 0.195, and 0.213%, respectively. Additionally, using ABT starter decreased the titratable acidity values of fresh yogurt and during storage time compared to yogurt manufactured with traditional culture [21]. The acidity development of ayran treatments during the post-acidification and cold storage was mainly due to the metabolic activity of probiotics [22].

**Table 1:** Acidity, moisture, and aroma compounds (acetaldehyde and diacetyl) of ayran

Properties	Treatments	Storage period (day)			
		1	7	14	Means ± SD
Acidity (%)	T1	0.85 ± 0.05	0.99 ± 0.01	1.07 ± 0.48	1.06 ± 0.32^A^
	T2	0.66 ± 0.11	0.74 ± 0.04	0.78 ± 0.02	0.73 ± 0.07^B^
	T3	0.76 ± 0.01	0.87 ± 0.03	0.92 ± 0.02	0.85 ± 0.07^B^
	T4	0.75 ± 0.05	0.85 ± 0.02	0.89 ± 0.01	0.83 ± 0.06^B^
	Means ± SD	0.75 ± 0.09^b^	0.88 ± 0.09^ab^	0.98 ± 0.30^a^	
Moisture (%)	T1	93.75 ± 2.08	93.72 ± 1.0	93.74 ± 3.0	93.51 ± 1.92^A^
	T2	93.62 ± 0.1	93.67 ± 1.0	93.59 ± 2.0	93.62 ± 1.12^A^
	T3	93.72 ± 1.0	93.70 ± 0.7	93.77 ± 0.4	93.78 ± 0.66^A^
	T4	93.66 ± 0.66	93.65 ± 0.05	93.62 ± 1.0	93.64 ± 0.59^A^
	Means ± SD	93.52 ± 1.05^a^	93.68 ± 0.67^a^	93.72 ± 1.61^a^	
Acetaldehyde (ppm)	T1	35.49 ± 2.0	21.06 ± 1.0	12.76 ± 0.7	23.10 ± 10.02^A^
	T2	29.98 ± 0.2	16.73 ± 1.0	8.29 ± 0.41	18.41 ± 9.38^B^
	T3	25.73 ± 0.02	11.96 ± 0.9	5.25 ± 0.25	14.31 ± 9.05^C^
	T4	27.49 ± 0.09	12.86 ± 0.2	7.35 ± 0.30	15.90 ± 9.02^D^
	Means ± SD	29.67 ± 3.93^a^	15.65 ± 3.82^b^	8.47 ± 2.88^c^	
Diacetyl (ppm)	T1	12.45 ± 0.05	15.50 ± 1.0	11.50 ± 0.2	13.11 ± 1.91^A^
	T2	9.07 ± 0.07	11.38 ± 1.0	18.80 ± 0.8	13.08 ± 4.45^A^
	T3	6.26 ± 1.0	7.02 ± 0.02	5.81 ± 0.1	6.36 ± 0.73^B^
	T4	7.75 ± 0.1	9.51 ± 0.30	6.16 ± 0.11	7.80 ± 1.47^B^
	Means ± SD	8.88 ± 2.43^a^	10.85 ± 3.29^a^	10.09 ± 5.51^a^	

*T1* ayran made using classic yogurt culture, *T2* ayran made using ABT-5 culture, *T3* ayran made using YO-Flex Mild 1.0 (EPS-producing) culture, *T4* ayran made using YO-Flex Mild 1.0 (EPS-producing) culture + BB-12

^abcd^Letters indication to significant differences between the samples of ayran ± SD

^ABCD^Letters indication to significant differences between the storage period of ayran ± SD

On the other side, the acidity levels of ayran prepared with EPS-producing culture [T3] were lower than that of control [T1] but higher than that of probiotic ayran [T2]. This could be related to the faster growth and acidification rates in classic culture (non-EPS strain) [23]. The addition of BB-12 to EPS-producing culture [T4] slightly reduced the ayran acidity compared with the acidity of EPS-producing culture ayran. Similar findings were previously found by Yilmaz et al. [7] about the titratable acidity values of ayran made using EPS-producing culture. They concluded that the highest acidity values in ayran samples were detected in the control group prepared using non-EPS strain as compared with those made utilizing EPS-producing *S. thermophilus* strains. Also, Swelam et al. [24] found that using EPS culture instead of classic culture decreased the acidity of yogurt from 0.74% (control sample) to 0.66%. In contrast to these outcomes, Behare et al. [6] stated that the lassi samples (drinkable yogurt) made by EPS-positive *S. thermophilus* showed higher titratable acidity than that made by EPS-negative *S. thermophiles*. In general, the findings of our study regarding ayran acidity agree with the previous findings [25], which found that the acidity of ayran samples was determined to be between 0.2 and 1.0%.

Data shown in Table 1 clearly indicate that using different cultures had no effect on the moisture contents of ayran samples. The moisture values were 93.75, 93.62, 93.72, and 93.66% for fresh ayran treatments produced with classic, probiotic, EPS-producing, and EPS-producing + BB-12 cultures, respectively. Also, insignificant variations in moisture amounts of ayran samples were detected within the storage period. The moisture contents in all ayran treatments were inside the range documented for the ayran food [26]. According to Ismail [21], the moisture levels in yogurt treatments produced by either traditional or ABT cultures were similar.

### Acetaldehyde and Diacetyl Contents of Ayran

As is well known, the yogurt culture bacteria *L. bulgaricus* and *S. thermophilus* produce acetaldehyde through a variety of different pathways, which gives yogurt its characteristic flavor. Table 1 shows the acetaldehyde and diacetyl concentrations of ayran samples. Using probiotic cultures had a significant (*P* < 0.05) effect on the acetaldehyde contents of ayran treatments. Using classic culture resulted in higher acetaldehyde content of ayran. The lowest acetaldehyde values were observed in EPS ayran samples. According to Bongers et al. [27], yogurt bacteria strains that do not produce EPS produce large amounts of acetaldehyde. Swelam et al. [23] reported that using a mixture of EPS-producing and yogurt cultures (1:1) in yogurt preparation produced the highest acetaldehyde content as compared with the control (yogurt culture), whereas the lowest content of acetaldehyde was recorded in EPS-producing culture yogurt. All ayran treatments showed a constant decrease in acetaldehyde levels throughout the period of storage, which may be related to lactic acid starter cultures’ ability to convert it to ethanol or diacetyl [24].

The diacetyl values of ayran samples followed the same trend as the acetaldehyde content. Control ayran possessed greater diacetyl levels than that of other treatments. The diacetyl content was in the following order: classic culture ayran > probiotic ayran > mixture culture ayran > EPS producing culture ayran. Diacetyl content gradually increased in various ayran samples up to the seventh day of storage before decreasing over the duration of the storage period.

### Ayran’s Fatty Acid Content

The influence of using different cultures on fatty acids profile of fresh ayran samples is illustrated in Tables 2 and 3.

**Table 2:** Effect of using different cultures on free fatty acids (%) content of fresh ayran

Fatty acids	*C*	Treatments			
		T1	T2	T3	T4
		Saturated fatty acids (SFA) %			
Butyric	4:0	2.7355	2.5989	2.0689	2.4524
Caproic	6:0	1.8854	1.8113	1.8073	1.7831
Caprylic	8:0	1.1207	1.0778	1.0787	1.0786
Capric	10:0	2.4519	2.3785	2.4130	2.3975
Undecanoic	11:0	0.3024	0.2842	0.2904	0.2872
Lauric	12:0	2.9172	2.7369	2.8888	2.8586
Tridecanoic	13:0	0.1811	0.1898	0.2083	0.2373
Myristic	14:0	11.4844	11.2406	11.5130	11.3776
Pentadecanoic	15:0	0.7207	0.7203	0.7303	0.7185
Palmitic	16:0	36.5232	36.1016	37.1632	36.6358
Heptadecanoic	17:0	0.8267	0.8442	0.8439	0.8300
Stearic	18:0	8.0830	8.0588	8.8073	8.7038
Arachidic	20:0	1.3813	1.2277	1.2419	1.4646
Behenic acid	22:0	1.0592	1.0533	0.1238	0.0463
	24:0	0.1576	0.0935	0.1048	0.1168
Total		71.8303	70.4174	71.2836	70.9881

Unsaturated fatty acids (USFA) %					
Myristioleic	14:1	1.4770	1.5534	1.4982	1.4659
	15:1	1.6267	1.6650	1.6380	1.6212
Palmitioleic	16:1	1.7676	1.8670	1.6677	1.6457
	17:1	0.4106	0.4747	0.4383	0.4309
Oleic	18:1	20.3174	21.2570	21.0612	21.4352
Linoleic	18:2	1.5225	1.6262	1.5747	1.6022
α-Linolenic	18:3	0.5773	0.5939	0.5811	0.5926
Gadoleic acid	20:1	0.1932	0.2491	0.2021	0.1598
	22:1	0.2817	0.3045	0.0561	0.0626
Total		28.1740	29.5908	28.7204	29.0161

*T1* ayran made using classic yogurt culture, *T2* ayran made using ABT-5 culture, *T3* ayran made using YO-Flex Mild 1.0 (EPS-producing) culture, *T4* ayran made using YO-Flex Mild 1.0 (EPS-producing) culture + BB-12

**Table 3:** Effect of using different cultures on free fatty acid indices ratios of fresh ayran

Treatments	SFA	USFA	MUSFA	PUSFA	SCFA	MCFA	LCFA
T1	71.8303	28.174	26.0742	2.0998	11.4131	53.7807	34.8062
T2	70.4174	29.5908	27.3707	2.2201	10.8876	53.3377	35.7747
T3	71.2836	28.7204	26.5926	2.1278	10.5471	54.4187	35.0342
T4	70.9881	29.0161	26.8963	2.1198	10.8574	53.602	35.5406

*SFA* saturated fatty acids, *USFA* unsaturated fatty acids, *MUFA* monounsaturated fatty acids (C:1), *PUSFA* polyunsaturated fatty acids (C:2 + C:3), *SCFA* short-chain fatty acids (C4 to C12), *MCFA* medium-chain fatty acids (C13 to C16), *LCFA* long-chain fatty acids (> C16). *T1* ayran made using classic yogurt culture, *T2* ayran made using ABT-5 culture, *T3* ayran made using YO-Flex Mild 1.0 (EPS-producing) culture, *T4* ayran made using YO-Flex Mild 1.0 (EPS-producing) culture + BB-12

### Saturated and Unsaturated Fatty Acids

Unsaturated fatty acid (USFA) levels were lower than saturated fatty acid (SFA) for various ayran treatments. Using ABT-5 in ayran making [T2] brought less SFA and more USFA. Also, ayran prepared by EPS-producing [T3] or EPS-producing + BB12 cultures [T4] had higher USFA and lower SFA values compared with the control sample [T1]. Levels of SFA were reduced by 1.97, 0.76, and 1.17%, whereas USFA increased by 5.03, 1.94, and 2.99% for samples T2, T3, and T4, respectively. These results agree with those reported by Caglayan et al. [28], who showed that probiotic Turkish yogurt had USFA values that were slightly higher than SFA as compared with whole one. Ismail et al. [29] stated that fresh Labneh prepared with ABT starter had significantly lower SFA and higher USFA contents than Labneh made with traditional culture.

USFA are generally recognized to be more necessary for human nutrition; the higher concentrations of USFA and lower concentrations of SFA observed in the ayran samples from our study improve the ayran’s nutrient value. N-3 PUFAs have a variety of health benefits against cardiovascular diseases, including anti-inflammatory and well-known hypotriglyceridemic effects [30].

All of the ayran samples contained palmitic acid (C16:0), myristic acid (C14:0), and stearic acid as the three most common acids between SFA (C18:0). For USFA, palmitoleic acid took second place to oleic acid as the dominant fatty acid.

### Monounsaturated and Polyunsaturated Fatty Acids

The total monounsaturated fatty acids value (MUSFA) of the probiotic ayran was 27.3707% (sample T2), as indicated in Table 3, which is higher than that of ayran made using classic starter (26.0742%, sample T1). The mixed culture ayran [T4] in the same context had greater MUSFA (26.8963%) than the control [T1], while using EPS-producing culture [T3] in ayran production led to a modest rise in MUSFA (26.5926%). As a consequence, MUSFA levels were increased by 4.94, 1.99, and 3.15% for treatments T2, T3, and T4, respectively. Polyunsaturated fatty acid (PUSFA) content exhibited the same pattern as MUSFA, where T2 and T4 possessed the highest ratios. PUSFA concentrations were raised by 5.72, 1.33, and 1.43% for treatments T2, T3, and T4, respectively. Overall, in various ayran treatments, MUSFA content was higher than PUSFA content. The dominant fatty acid of MUSFA in all ayran samples was oleic acid. Linoleic acid represented as the PUSFA’s major fatty acid.

Among the essential fatty acids, omega fatty acids are essential to human health. α-linolenic acid (omega-3), oleic acid (omega-9), and linoleic acid (omega-6) concentrations increased in ayran prepared with probiotic or mixed cultures. Increasing rates were 4.62, 3.66, and 5.50% for oleic acid, 6.8, 3.4, and 5.23% for linoleic acid, and 2.87, 0.65, and 2.65% for α-linolenic acid in samples T2, T3, and T4, respectively. Numerous authors have discussed the benefits of linoleic and α-linolenic acids for health. Simopoulos [31] showed that the first discovery of the health benefits of omega-3 fatty acids, eicosa pentaenoic acid (EPA), and docosa hexaenoic acid (DHA) was in the Greenland Eskimos, as a result of their consumption of high amounts of seafood rich in the previous acids, which reduce the incidence of asthma, coronary heart disease, multiple sclerosis, and type 1 diabetes mellitus. Since that discovery, studies have revealed that omega-3 fatty acids are also effective against cancer, inflammatory bowel disease, rheumatoid arthritis, psoriasis, and rheumatoid factors. Our results are consistent with those reported by Paszczyk and Tonska [32]; they illustrated that utilizing cultures enriched with *Bifidobacterium bifidum* (BB-12) in yogurt preparation increased the content of conjugated linolenic acid compared with that made using cultures free from BB-12.

### Short-Chain Fatty Acids (C4–C12)

The amount of short-chain fatty acids (SCFA) in the ayran samples decreased when probiotic, EPS-producing, or EPS-producing + BB12 cultures were used as indicated in Table 3. The most common SCFA in all of the ayran samples was the fatty acid lauric (C:12), followed by butyric acid (C4) and capric acid (10). According to Ghoneem et al. [33], ABT-Labneh has lower SCFA contents than that produced with traditional culture. Production of volatile free fatty acids (C2-C10) was more active in the mixed yogurt cultures than in the pure ones due to the stimulating impact of protocol-operation between the two thermophilic species on the metabolic activities that are responsible for the production of free fatty acids.

### Medium-Chain Fatty Acids (C13–C16)

Medium-chain fatty acids (MCFA) were presented in slightly higher concentrations in the EPS-producing ayran [T3] compared to other ayran treatments. Palmitic acid (C16) had the highest value of MCFA, and myristic acid (C14) had the second-highest value.

### Long-Chain Fatty Acids (> C16)

Ayran produced using classic culture had lower concentrations of long-chain fatty acids (LCFA) than ayran produced using probiotics and mixed cultures. Oleic acid (C18:1) was the most prevalent LCFA acid in all the ayran samples, followed by stearic acid (C18).

### Antioxidant Activity of Ayran During Storage Period

The levels of antioxidant activity of fresh ayran are listed in Table 4. The antioxidant activity values were markedly higher in probiotic ayran [T2] than in the control [T1]. These results are in line with those presented by Wang et al. [34], who showed that probiotics have antioxidant activity by secreting enzymes like superoxide dismutase (SOD) and stimulating the formation of glutathione, a major non-enzymatic antioxidant and free radical scavenger (GSH). Superoxide is catalyzed by SOD into water and hydrogen peroxide. By collaborating with selenium-dependent GSH peroxidase, GSH eliminates radicals such as hydroxyl radicals and hydrogen peroxides. Additionally, probiotics stimulate the production of certain antioxidant biomolecules, including exopolysaccharides [35]. Çakmakçı et al. [36] revealed that yogurt produced with classic culture + *Lactobacillus acidophilus* had higher antioxidant properties than that of control made using classic culture.

**Table 4:** Antioxidant activity of ayran during storage

Treatments	Storage period (day)			
	1	7	14	Means ± SD
T1	6.378 ± 1.0	9.185 ± 0.18	7.126 ± 0.12	7.55 ± 1.35^D^
T2	9.919 ± 0.2	12.365 ± 0.3	10.338 ± 1.0	10.86 ± 1.14^C^
T3	13.588 ± 0.5	16.458 ± 3.0	14.254 ± 0.2	14.53 ± 1.83^B^
T4	23.272 ± 1.0	31.268 ± 0.26	28.348 ± 0.3	27.62 ± 3.54^A^
Means ± SD	13.28 ± 6.61^c^	17.14 ± 8.95^a^	15.01 ± 8.0^b^	

*T1* ayran made using classic yogurt culture, *T2* ayran made using ABT-5 culture, *T3* ayran made using YO-Flex Mild 1.0 (EPS-producing) culture, *T4* ayran made using YO-Flex Mild 1.0 (EPS-producing) culture + BB-12

^abcd^Letters indication to significant differences between the samples of ayran ± SD

^ABCD^Letters indication to significant differences between the storage period of ayran ± SD

Further increases in antioxidant activity were achieved by using EPS-producing culture in ayran preparation [T3]. Wang et al. [37] reported that an EPS from the *L. plantarum KX041* strain exhibited high antioxidant activity with the ability to scavenge of free radicals for the following sources: DPPH, 2,2-azinobis (3-ethylbenzthiazoline)-6-sulfonic acid (ABTS), super-oxide free radicals, and hydroxyl. According to Yamamoto et al. [38], exopolysaccharides, isoflavone aglycones, and antioxidant peptides may be the antioxidant substances produced by LAB fermentation. High antioxidant capacity was exhibited by an acidic exopolysaccharide produced by *Pediococcus pentosaceus* MYU 759.

Ayran made using EPS-producing + BB12 culture (sample T4) was highly richer in the value of DPPH inhibition than other treatments which may be attributed to the activity of probiotic (BB12) and their cooperative relationship with EPS-producing culture. In treatments T2, T3, and T4, the rates of increase in the antioxidant activity values of fresh ayran were 55.52, 113.04, and 264.88%, respectively. The high antioxidant activity in mixed culture ayran no doubt increases its health importance. Xu et al. [39] reported that acidic EPS and neutral EPS produced by *Bifidobacterium animalis* RH showed antioxidant activity in vitro and in vivo. In a galactose-induced aged mouse model, oral administration of the EPSs of *B. animalis* RH significantly increased the activities of antioxidant enzymes such as SOD and catalase (CAT), the total antioxidant capacity in serums, and glutathione S-transferase (GST) in the liver.

The antioxidant activity values in various ayran treatments gradually increased to the seventh day of storage, at which point they started to decline. The increase in degradation of phenolic compounds with antioxidant activities and/or the increase in milk protein polyphenol interaction may be responsible for the decrease in antioxidant activities during cold storage of yogurt [40]. On the contrary, Akkoyun and Arslan [41] showed that the total antioxidant activity gradually increased during the refrigerated storage period (14 days) of ayran.

### Cholesterol Content of Ayran

**Fig. 1:**
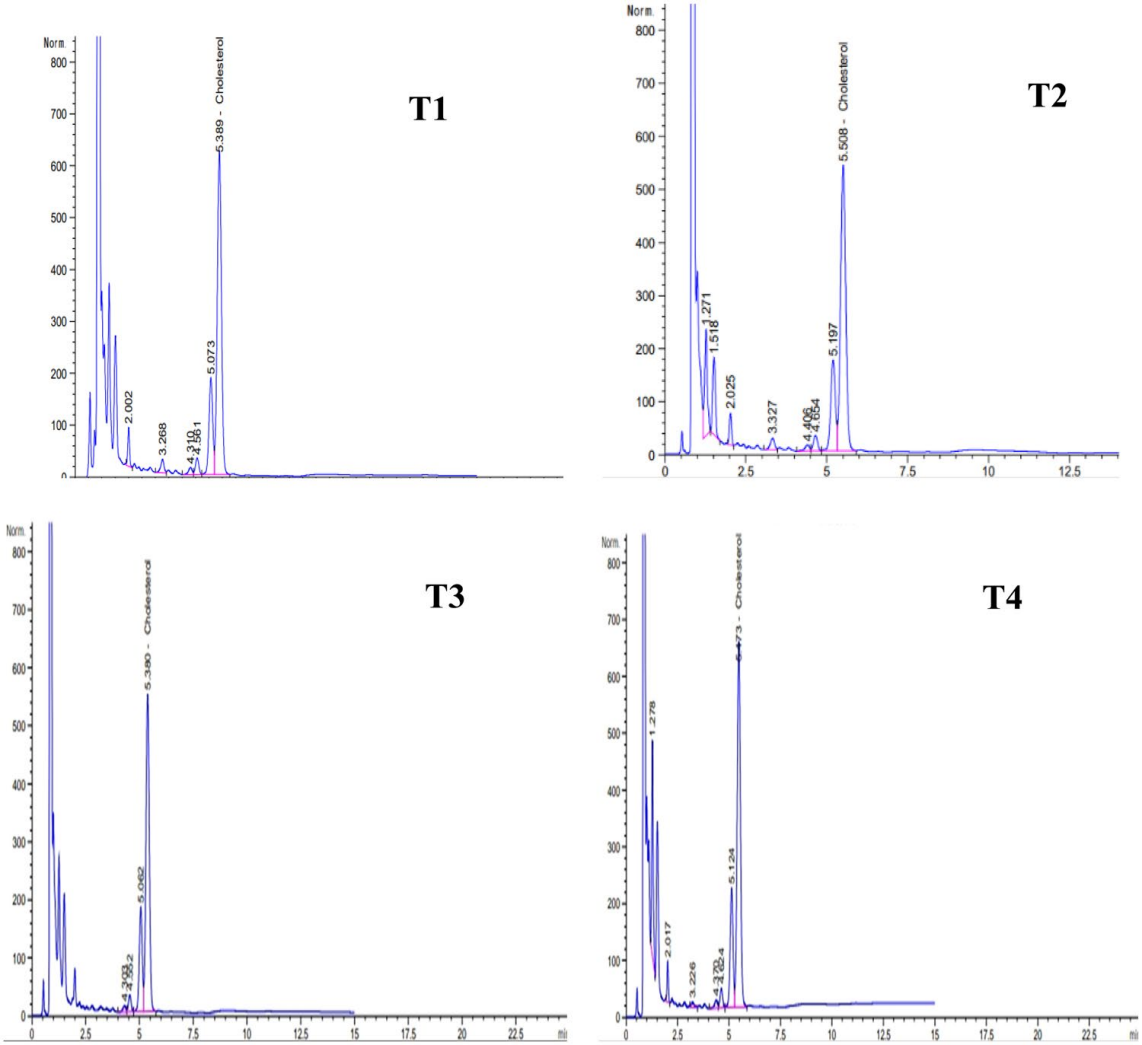
Cholesterol profiles of various fresh ayran samples

Data presented in Table 5 and Fig. 1 show the cholesterol amounts of fresh ayran samples. The data indicate that the control sample [T1] had the highest cholesterol level followed by EPS-producing culture ayran [T3]. Cultures containing probiotic (ABT-5 or BB-12) reduced the cholesterol values in the ayran produced (T2 and T4). The cholesterol concentrations of samples T1, T2, T3, and T4 were 11.188, 9.004, 11.180, and 8.983 mg/100 g, respectively. In similar findings to our current study, according to Albano et al. [42], cholesterol-lowering activity is one of the most promising properties of lactic acid bacteria (LAB) with probiotic characteristics. LAB also represent a potential method for lowering the cholesterol content of food. However, to date, very few attempts have been undertaken to use LAB to reduce the cholesterol in foods, notably in dairy products, as a potential substitute for more expensive chemical and physical processes, which can change the texture of food and remove flavor. Additionally, they claimed that seven probiotic lactic acid bacteria strains (*Lactobacillus paracasei* ssp. *paracasei* SE160 and VC213, *Lactobacillus plantarum* VS166 and VS513, *Lactobacillus casei* VC199, *Enterococcus lactis* BT161, and *Enterococcus faecium* VC223) had the capability to reduce cholesterol in cheese. However, Paszczyk and Tonska [32] reported that the levels of atherogenicity index, index thrombogenicity, and hypocholesterolemic/hypercholesterolemic (the lipid quality indices) were similar in yogurt sample produced by cultures enriched with or without BB-12.

**Table 5:** Cholesterol and folic acid profiles of various fresh ayran samples

Properties	Treatments	Retention time (min)	Peak area (mAU*s)	Concentration (mg/100 g)
Cholesterol	T1 T2 T3 T4	5.389 5.508 5.473 5.383	6801.994 6013.703 7161.807 5996.495	11.188 9.004 11.180 8.983
Folic acid	T1 T2 T3 T4	8.674 8.685 8.676 8.575	3.27098 3.55775 3.29994 3.73046	0.1050 0.1453 0.1348 0.1566

*T1* ayran made using classic yogurt culture, *T2* ayran made using ABT-5 culture, *T3* ayran made using YO-Flex Mild 1.0 (EPS-producing) culture, *T4* ayran made using YO-Flex Mild 1.0 (EPS-producing) culture + BB-12

### Folic Acid Content of Ayran

**Fig. 2:**
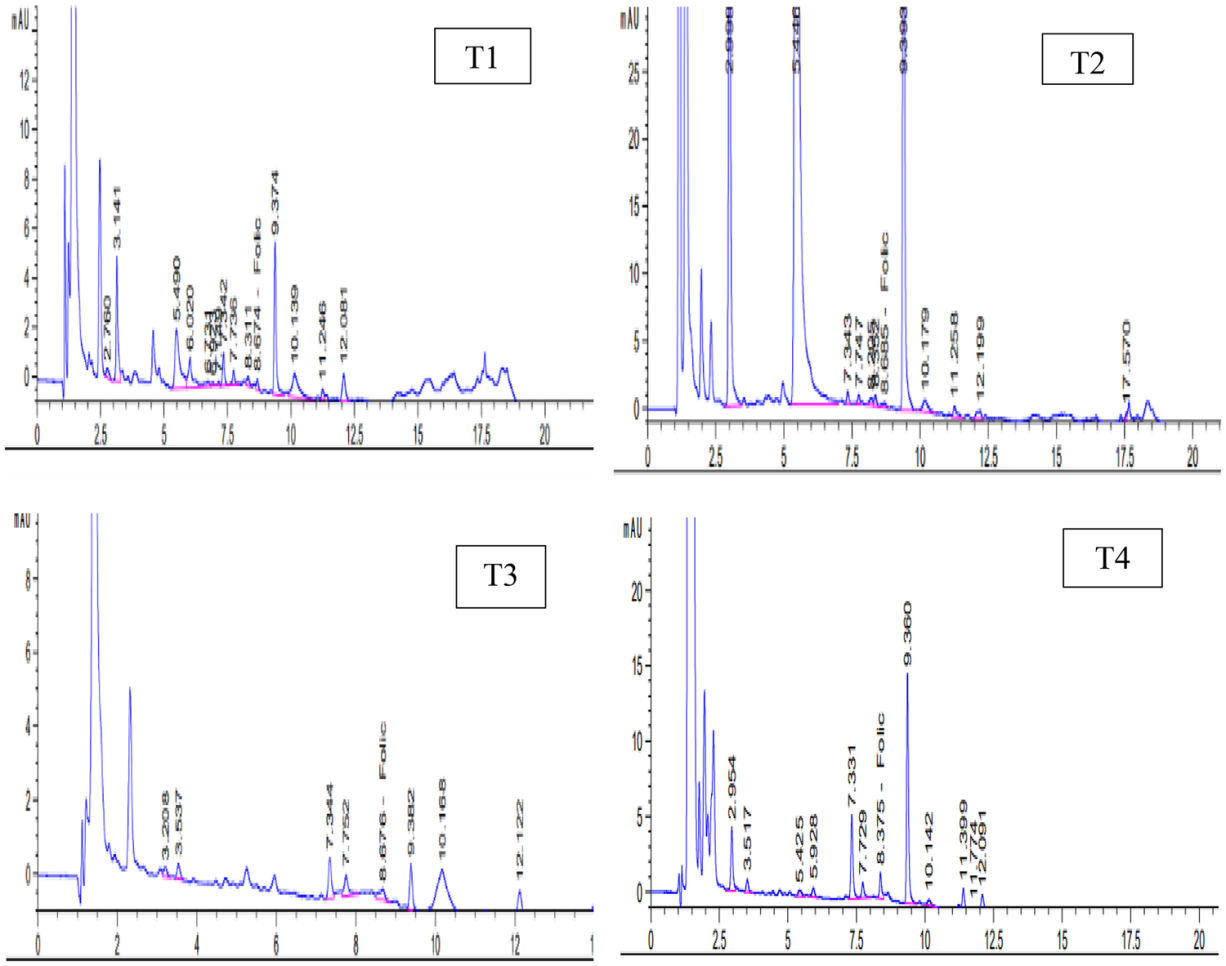
Folic acid profiles of various fresh ayran samples

Due to lactic acid bacteria, fermented milk products have been shown to provide 2–4 times more folate than raw milk. As a result, lactic acid bacteria can be used as an effective probiotic to treat a deficiency of folate because they can survive in the gastrointestinal tract and produce a high amount of folate [43]. The folic acid content of ayran made using different cultures is shown in Table 5 and Fig. 2. The folic acid amounts were increased with using probiotic [T2] or BB-12 [T4] cultures in ayran preparation. The largest folic acid level was recorded for the mixture culture ayran (0.1566 mg/100 g) followed by probiotic one (0.1453 mg/100 g), whereas the control sample [T1] had the lowest value (0.1050 mg/100 g). Crittenden et al. [44] reported that a six-fold increase in folate in the skim milk medium was recorded when two folate-producing organisms (*Bifidobacterium animalis* CSCC 1941 and *Streptococcus thermophiles* CSCC 2000) were used. Rossi et al. [45] found that when comparing probiotic yogurt, original milk, and conventional fermented milk, the use of folate-producing probiotic bacteria in combination with *L. bulgaricus* and/or *S. thermophilus* results in the largest increase in folate.

## Conclusions

The results of our study indicate that the type of culture utilized to manufacture yogurt drink ayran significantly affected the chemical composition. Using EPS-producing culture + *Bifidobacterium animalis* subsp. *Lactis* (BB12) led to a significant decrease in cholesterol level and a significant increase in monounsaturated, polyunsaturated fatty acids, oleic acid, linoleic acid, α-linolenic acid, antioxidant activity, and folic acid of ayran. These effects, of course, will be reflected in the nutritional and health value of this product. Therefore, we recommend using these mixed probiotic cultures to produce ayran with a high health value. Also, it is recommended to consume this product, particularly during the summer and in countries with hot climate.

## Data Availability

All data obtained during this study are included in the article.
